# CT-based deep learning radiomics nomogram for the prediction of pathological grade in bladder cancer: a multicenter study

**DOI:** 10.1186/s40644-023-00609-z

**Published:** 2023-09-18

**Authors:** Hongzheng Song, Shifeng Yang, Boyang Yu, Na Li, Yonghua Huang, Rui Sun, Bo Wang, Pei Nie, Feng Hou, Chencui Huang, Meng Zhang, Hexiang Wang

**Affiliations:** 1 Department of Radiology, The Affiliated Hospital of Qingdao University, 16 Jiangsu Road, Qingdao, Shandong China; 2 Department of Radiology, Shandong Provincial Hospital Affiliated to Shandong First Medical University, Jinan, Shandong China; 3 Qingdao No.58 High School of Shandong Province, Qingdao, Shandong China; 4 Department of Radiology, The People’s Hospital of Zhangqiu Area, Jinan, Shandong China; 5 Department of Radiology, The Puyang Oilfield General Hospital, Puyang, Henan China; 6 Department of Pathology, The Affiliated Hospital of Qingdao University, Qingdao, Shandong China; 7 Department of Research Collaboration, R&D Center, Beijing Deepwise & League of PHD Technology Co., Ltd, Beijing, China

**Keywords:** Urinary bladder neoplasms, Computed tomography, Deep learning, Radiomics, Nomogram

## Abstract

**Background:**

To construct and assess a computed tomography (CT)-based deep learning radiomics nomogram (DLRN) for predicting the pathological grade of bladder cancer (BCa) preoperatively.

**Methods:**

We retrospectively enrolled 688 patients with BCa (469 in the training cohort, 219 in the external test cohort) who underwent surgical resection. We extracted handcrafted radiomics (HCR) features and deep learning (DL) features from three-phase CT images (including corticomedullary-phase [C-phase], nephrographic-phase [N-phase] and excretory-phase [E-phase]). We constructed predictive models using 11 machine learning classifiers, and we developed a DLRN by combining the radiomic signature with clinical factors. We assessed performance and clinical utility of the models with reference to the area under the curve (AUC), calibration curve, and decision curve analysis (DCA).

**Results:**

The support vector machine (SVM) classifier model based on HCR and DL combined features was the best radiomic signature, with AUC values of 0.953 and 0.943 in the training cohort and the external test cohort, respectively. The AUC values of the clinical model in the training cohort and the external test cohort were 0.752 and 0.745, respectively. DLRN performed well on both data cohorts (training cohort: AUC = 0.961; external test cohort: AUC = 0.947), and outperformed the clinical model and the optimal radiomic signature.

**Conclusion:**

The proposed CT-based DLRN showed good diagnostic capability in distinguishing between high and low grade BCa.

**Supplementary Information:**

The online version contains supplementary material available at 10.1186/s40644-023-00609-z.

## Introduction

Bladder cancer (BCa) is the tenth most commonly diagnosed cancer worldwide, and is more common in men than in women [1, 2]. Tumor grade is a crucial prognostic indicator in BCa [3], and has a major impact on treatment decisions and prognosis. High-grade BCa has a higher rate of progression and recurrence [3–5]. The progression and recurrence rates for low-grade tumors were 4% and 43%, respectively. Corresponding values for high-grade tumors were 19% and 58% [3]. Patients with low-grade tumors usually undergo transurethral resection of bladder tumor (TURBT) [6], while high-grade tumors patients present a higher risk of progression and recurrence after TURBT [7], and may need to consider partial or radical cystectomy [6, 8]. For the above reasons, preoperative determination of pathological grade is essential for patients with BCa.

Currently, cystoscopic resection and biopsy still represent the standard methods for grading BCa [9]. Typical results from biopsy, however, may lead to diagnostic errors attributable to inadequate specimens and tumor heterogeneity [10, 11]. Repeated examinations could improve the accuracy of the diagnosis, but this procedure is undesirable because of its invasive nature and carries substantial risks of bladder perforation [12]. The development of a non-invasive preoperative evaluation method would greatly benefit the management of patients with BCa.

Computed tomography (CT) examination is a common method for the preoperative evaluation of BCa patients. However, tumor heterogeneity cannot be reliably assessed with the naked eye [13]. Radiomics is an image analysis approach that can extract a large number of quantitative features from medical images, and that supports quantitative expression of tumor heterogeneity [14]. Recently, it has attracted much attention in predicting tumor stage, pathological grade, lymph node metastasis, muscle-invasive status, and therapeutic response associated with BCa [13, 15–19].

Deep learning (DL) is an emerging technology with great promise, which can build a model using effective feature data extracted from images to reflect the correlation between image information and specific diseases [18]. This model-based technology can improve the prediction of disease characteristics [20]. CT-based DL models implemented by convolutional neural networks (CNN) have shown great potential in evaluating treatment response, and in predicting muscular invasiveness of BCa [18, 19]. However, there are no reports on CT-based DL models for the prediction of BCa grade using data from a multicenter study. To address this lack of knowledge in the literature, we constructed a CT-based DL radiomics nomogram (DLRN) to predict the histopathological grade of BCa preoperatively.

## Patients and methods

### Patients

The review boards of all participating institutions approved this retrospective study. The requirement for patient informed consent was waived. This study used data from three medical centers. We collected BCa patients confirmed by pathology who had undergone surgical resection between October 2014 and December 2022. The inclusion criteria were: (1) pathologically confirmed BCa grading; (2) CT urography (CTU) during the 30 days preceding surgery. Patients were excluded if: (1) they received preoperative treatment, including chemotherapy, radiotherapy, or immunotherapy; (2) they had more than one lesion; (3) their preoperative CT image was of poor quality; (4) their clinicopathological dataset was incomplete; (5) they suffered from other concomitant tumor disease.

We collected 688 patients for this study. Of these, 469 patients from the Affiliated Hospital of Qingdao University were assigned to the training cohort, and 219 patients from the Shandong Provincial Hospital Affiliated to Shandong First Medical University and from Puyang Oilfield General Hospital were assigned to the external test cohort.

### CT examinations

All enrolled patients underwent CTU examinations. Table S1 details the settings adopted for CT scanning. Enhanced scans acquired images of corticomedullary-phase (C-phase), nephrographic-phase (N-phase), and excretory-phase (E-phase) at (respectively) 25 s, 75 s, and 300 s after the bolus-triggering threshold of 120 HU was reached in the thoracoabdominal aorta junction.

### Collection and evaluation of clinical and CT features

We analyzed both clinical and CT features obtained from patients, including age, gender, TNM stage, size, shape, boundary, calcification, cystic necrosis, stalk, extramural infiltration, regional lymph node metastasis, lesion CT value in corticomedullary-phase (LCTV-C), lesion CT value in nephrographic-phase (LCTV-N), and lesion CT value in excretory-phase (LCTV-E). Three radiologists, who were unaware of the pathological findings before the evaluation, separately assessed CT features from CT images. In case of disagreement, consensus was reached by consultation.

### Segmentation of region of interest and extraction of radiomics features

**Fig. 1:**
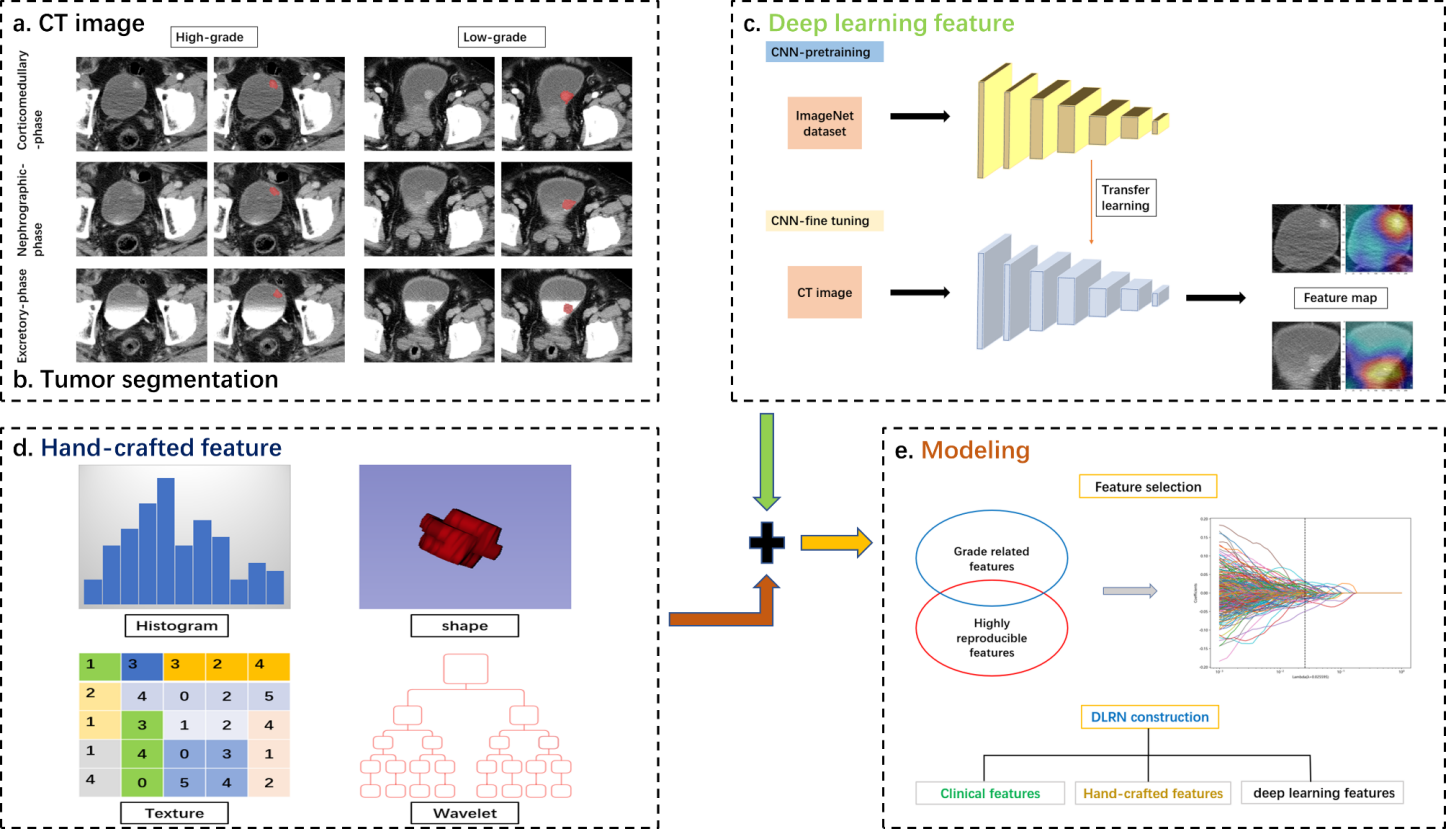
Flow chart of this study

Figure 1 shows a flow chart of this study. Two radiologists, who were unaware of the pathological findings, manually and independently delineated the region of interest (ROI) on the three-phase images using ITK-SNAP software (version 3.8.0, http://www.itksnap.org). We randomly selected 94 lesions to be re-segmented by the two radiologists, and the same procedure was repeated by one of the radiologists 3 weeks later to assess intra- and interclass correlation coefficients (ICCs).

We used the Python package Pyradiomics (version 3.0.1) to extract handcrafted radiomics (HCR) features from ROI. High-pass or low-pass wavelet filters and Laplacian of Gaussian filter with different λ parameters were used to preprocess the original images to enhance the recognition of features. Based on original and preprocessed images, we obtained 3948 HCR features from the ROIs of the three-phase images, including 756 first-order features, 42 shape-based features and texture features, including1008 gray-level co-occurrence matrix (glcm) features, 672 Gy-level run-length matrix (glrlm) features, 672 Gy-level size zone matrix (glszm) features, 588 Gy-level dependence matrix (gldm) features, 210 neighboring grey tone difference matrix (ngtdm) features, and among them, there are 2232 wavelet-based features.

We selected the ResNet18 network pre-trained on the ImageNet database for DL-based feature extraction. The original images were resized to 224 × 224 pixels before training. The model was trained using the stochastic gradient descent optimizer with an initial learning rate of 3 × 10^− 4^, decayed by the cosine annealing algorithm for 22 epochs, and a batch size of 256. We used the fixed network parameters after model training as feature extractors. More specifically, we used the output of the penultimate layer of the trained CNN to define DL features. We trained the model separately on three-phase images using the same approach. ResNet18 extracted 1536 features from the ROIs of the three-phase images for each patient.

### Image normalization methodology

We adopted a combat compensation methodology to retain the specific characteristics of texture patterns. This approach removed differences in radiomic features associated with different scanners, scanning protocols, and parameter settings [21]. In this study, we used combat compensation methodology to reduce the inconsistencies of multi-center radiomic features. We then normalized all features via Z scoring.

### Feature selection and construction of machine learning models

To reduce the dimensionality of high-dimensional features, we adopted the following procedure. First, our analysis only retained HCR features for which ICCs were > 0.8. Second, we screen features using the least absolute shrinkage and selection operator (LASSO) algorithm. Finally, we constructed predictive models based on HCR features, DL features. We construct machine learning models using 11 machine learning classifiers, including logistic regression (LR), NaiveBayes, support vector machine (SVM), K nearest neighbor (KNN), Light Gradient Boosting Machine (LightGBM), RandomForest, eXtreme Gradient Boosting (XGBoost), GradientBoosting, extremely randomized trees (ExtraTrees), AdaBoost, and Multi-Layer perceptron (MLP). We adopted the 10-fold cross-validation approach to train classifiers on the training cohort. To evaluate model capability for grading BCa, we relied on the area under the receiver operating characteristic (ROC) curve (AUC) and on accuracy. Based on these metrics, we selected the best machine learning model.

### Clinical model and nomogram construction

We used univariate logistic regression to select clinical and CT features associated with BCa grading. We included variables with *p <* 0.05 for multivariate logistic regression. We then built a clinical model using features with *p* < 0.05 of multivariate logistic regression. We combined factors from the clinical model with the radiomic signature of the best machine learning model to build a DLRN. We used the likelihood ratio test based on the Akaike information criterion to identify factors associated with BCa grading. We used the ROC curve to evaluate the diagnostic capability of both clinical model and DLRN applied to the two data cohorts. We inspected calibration curves to judge goodness of fit of the DLRN. We assessed clinical utility using decision curve analysis (DCA). We applied the DeLong test to compare differences in AUC values across models.

### Follow-up surveillance

All patients were followed up postoperatively every 3–6 months during the first 2 years, and annually thereafter. The duration of progression-free survival (PFS) was defined as the time between patients undergoing surgery and detection of local recurrence, the last follow-up, or death. The deadline for follow-up was January 12, 2023.

### Statistical analysis

We used SPSS software (version 26.0, IBM) to analyze the differences in clinical and CT characteristics of patients. Python (version 3.9.7, www.python.org) was used for building machine learning models. The model performance was evaluated using R software (version 4.2.2, www.r-project.org). To compare clinical and CT data between the two cohorts, we applied Mann-Whitney *U* tests, Fisher’s exact tests, or Chi-squared tests as appropriate. We adopted *p <* 0.05 to define statistically significant differences. We relied on the Kaplan-Meier method and log-rank tests to evaluate the PFS probability of patients in the different risk groups.

## Results

### Clinical and CT characteristics

Table 1 lists clinical and CT characteristics of patients in the two data cohorts. We measured significant differences in gender, T stage, shape, size, boundary, stalk, LCTV-C, LCTV-N, and LCTV-E between the two cohorts. The remaining characteristics were not statistically significant between the two cohorts.

**Table 1:** Clinical and CT characteristics for patients

Characteristics	Training cohort (n = 469)	External test cohort (n = 219)	*p* value
Age (y) Median (IQR)	66(59–74)	65(58–72)	0.083
Gender Male Female	469(100.0) 0(0.0)	175(79.9) 44(20.1)	<0.001
Grade Low grade High grade	170(36.2) 299(63.8)	87(39.7) 132(60.3)	0.380
T stage ≤ T1 >T1	306(65.2) 163(34.8)	170(77.6) 49(22.4)	0.001
N stage N0 N1 N2 N3	436(93.0) 22(4.7) 8(1.7) 3(0.6)	213(97.3) 4(1.8) 1(0.5) 1(0.5)	0.137
M stage M0 M1	466(99.4) 3(0.6)	219(100.0) 0(0.0)	0.555
Shape Cauliflower Papillary Irregular	228(48.6) 116(24.7) 125(26.7)	167(76.3) 33(15.1) 19(8.7)	<0.001
Size (cm) Median (IQR)	3.0(1.9–4.5)	2.4(1.7–3.4)	<0.001
Calcification No Yes	395(84.2) 74(15.8)	186(84.9) 33(15.1)	0.811
Cystic necrosis No Yes	429(91.5) 40(8.5)	195(89.0) 24(11.0)	0.307
Boundary No Yes	186(39.7) 283(60.3)	7(3.2) 212(96.8)	<0.001
Stalk No Yes	375(80.0) 94(20.0)	81(37.0) 138(63.0)	<0.001
Extramural infiltration No Yes	412(87.8) 57(12.2)	195(89.0) 24(11.0)	0.651
RLN metastasis No Yes	446(95.1) 23(4.9)	214(97.7) 5(2.3)	0.105
LCTV-C (HU) Median (IQR)	58.6(47.0–73.0)	67.0(58.0–81.0)	<0.001
LCTV-N (HU) Median (IQR)	73.0(63.0-86.3)	75.0(65.0–91.0)	0.029
LCTV-E (HU) Median (IQR)	72.1(62.0–85.0)	76.0(63.0–95.0)	0.006

IQR, interquartile; RLN, reginal lymph node; LCTV-C, lesion CT value in corticomedullary-phase; LCTV-N, lesion CT value in nephrographic-phase; LCTV-E, lesion CT value in excretory-phase

### Construction of clinical model

Table 2 lists positive results for clinical and CT characteristics from univariate and multivariate logistic regression analysis. Multivariate logistic regression identified age, size, shape, boundary, stalk, and extramural infiltration as independent predictors of BCa grade. AUC values for the clinical model applied to training and external test groups were 0.752 and 0.745, respectively.

**Table 2:** Positive results of univariate and multivariate logistic regression for clinical and CT characteristics in patients

Variable	Univariate		Multivariate	
	OR (95% CI)	*p value*	OR (95% CI)	*p* value
Age	1.038(1.020–1.057)	<0.001	1.032(1.012–1.051)	0.001
Size	1.408(1.253–1.582)	<0.001	1.277(1.119–1.457)	<0.001
Shape	1.374(1.091–1.732)	0.007	1.565(1.188–2.062)	0.001
Boundary	0.618(0.421–0.906)	0.014	0.503(0.324–0.780)	0.002
Cystic necrosis	3.507(1.441–8.535)	0.006	1.058(0.378–2.964)	0.914
Stalk	0.414(0.262–0.656)	<0.001	0.575(0.341–0.969)	0.038
Extramural infiltration	8.941(3.176–25.175)	<0.001	4.296(1.369–13.479)	0.012
RLN metastasis	13.422(1.793-100.489)	0.011	5.819(0.696–48.658)	0.104
LCT V-C	1.012(1.003–1.021)	0.010	1.009(0.997–1.020)	0.143
LCTV-N	1.015(1.004–1.026)	0.006	1.010(0.995–1.025)	0.198
LCTV-E	1.011(1.002–1.021)	0.020	0.999(0.988–1.010)	0.905

OR, odds ratio; CI, confidence interval; RLN, reginal lymph node; LCTV-C, lesion CT value in corticomedullary-phase; LCTV-N, lesion CT value in nephrographic-phase; LCTV-E, lesion CT value in excretory-phase

### Construction and evaluation of radiomic signature

**Fig. 2:**
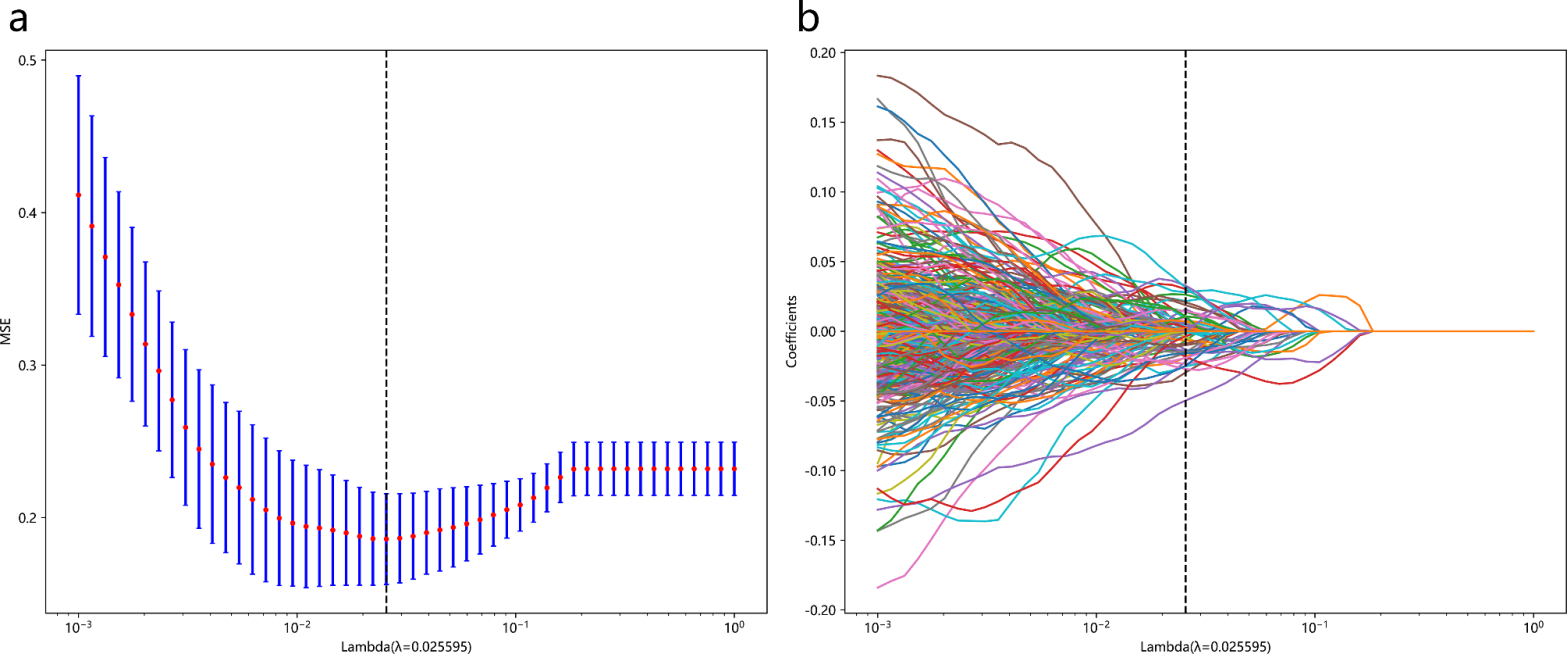
Feature screening utilizing LASSO algorithm. The cross-validation plot **(a)** and the coefficient profile plot **(b)**

**Fig. 3:**
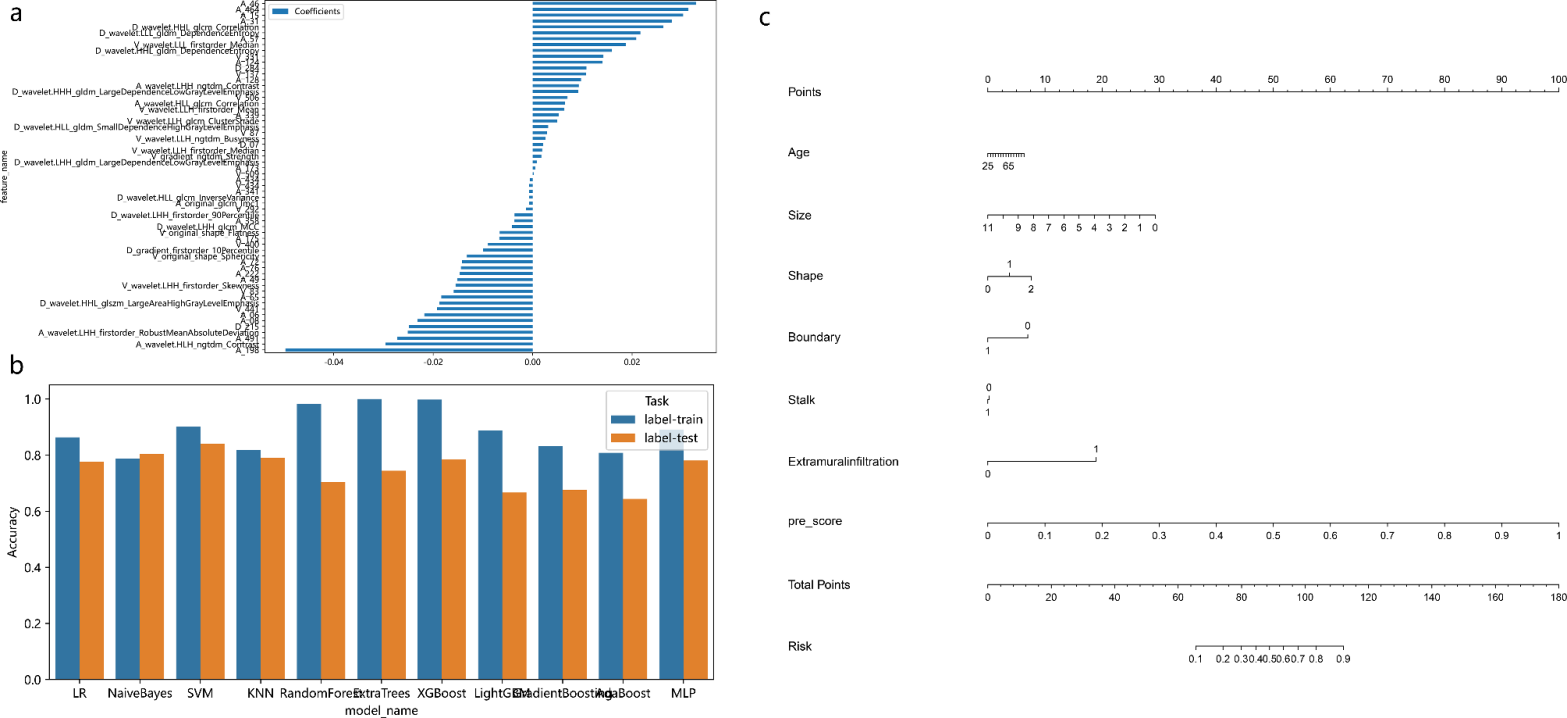
Feature weight histogram of the features used in this study **(a)**. Accuracy histogram of different machine learning models based on DLR **(b)**. Nomogram of the training cohort **(c)**

We retained 2636 HCR features with ICCs > 0.80. We combined these features with 1536 DL features for subsequent analysis, and reduced their dimensionality using the LASSO algorithm (Fig. 2). We selected the 60 most significant features and incorporated them into the model combining DL features and HCR features (DLR model). These features included 25 HCR features and 35 DL features. Of these, the DL features carried largest predictive weight (Fig. 3a). We constructed predictive models using 11 machine learning algorithms. Table 3 details the predictive performance of the DLR-based signature for identifying pathological grade of BCa. Table S2 provides additional details on prediction via HCR features and DL features. The MLP classifier model produced the best predictive performance with reference to the HCR signature (AUC values of 0.850 and 0.848, and accuracy values of 0.780 and 0.790, for training and external test groups, respectively). The NaiveBayes classifier model produced the best predictive performance with reference to the DL signature (AUC values of 0.843 and 0.882, and accuracy values of 0.778 and 0.813, for training and external test groups, respectively). When applied to the external test group, the DLR-based SVM classifier outperformed the signatures based on HCR features and DL features. This was the best machine learning model, with an AUC value of 0.943 and an accuracy of 0.840 (Table 3; Fig. 3b**)**. A DeLong test indicated that the AUC values for the DLR-based SVM classifier and the clinical model were significantly different between the two cohorts (training cohort: *p* < 0.001, external test cohort: *p* = 0.042).

**Table 3:** Performance of different machine learning algorithms with reference to the DLR signature

	Training cohort		External test cohort	
	AUC (95% CI)	Accuracy	AUC (95% CI)	Accuracy
LR	0.931(0.909–0.954)	0.864	0.856(0.805–0.907)	0.776
NaiveBayes	0.854(0.819–0.889)	0.787	0.884(0.836–0.932)	0.804
SVM	0.953(0.931–0.974)	0.902	0.943(0.916–0.970)	0.840
KNN	0.899(0.873–0.925)	0.819	0.799(0.740–0.859)	0.790
RandomForest	0.999(0.997-1.000)	0.983	0.778(0.718–0.837)	0.703
ExtraTrees	1.000(nan-nan)	1.000	0.828(0.775–0.881)	0.744
XGBoost	1.000(nan-nan)	0.998	0.850(0.796–0.904)	0.785
LightGBM	0.986(0.979–0.993)	0.887	0.821(0.766–0.875)	0.667
GradientBoosting	0.936(0.915–0.958)	0.832	0.817(0.756–0.877)	0.676
AdaBoost	0.872(0.840–0.904)	0.808	0.636(0.561–0.711)	0.644
MLP	0.959(0.943–0.975)	0.891	0.878(0.835–0.922)	0.781

AUC, area under the curve; CI, confidence interval; LR, logistic regression; SVM, support vector machine; KNN, K nearest neighbor; ExtraTrees, extremely randomized trees; XGBoost, eXtreme Gradient Boosting; LightGBM, Light Gradient Boosting Machine; MLP, Multi-Layer perceptron

### Evaluation of nomogram and patient risk stratification

**Fig. 4:**
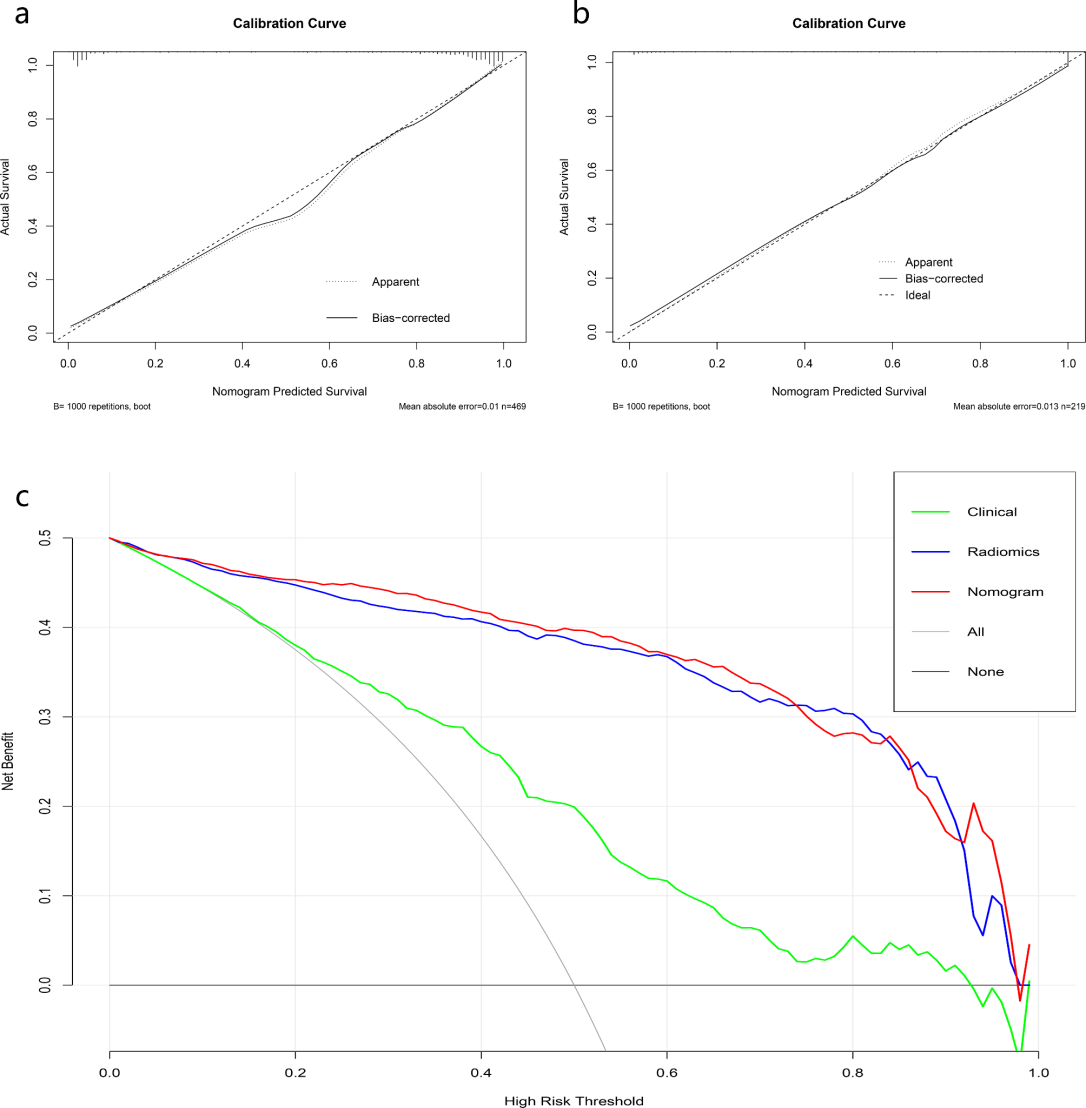
Calibration curves **(a, b)** of deep learning radiomics nomogram for training and external test cohorts. Decision curve analysis for different models **(c)**

**Fig. 5:**
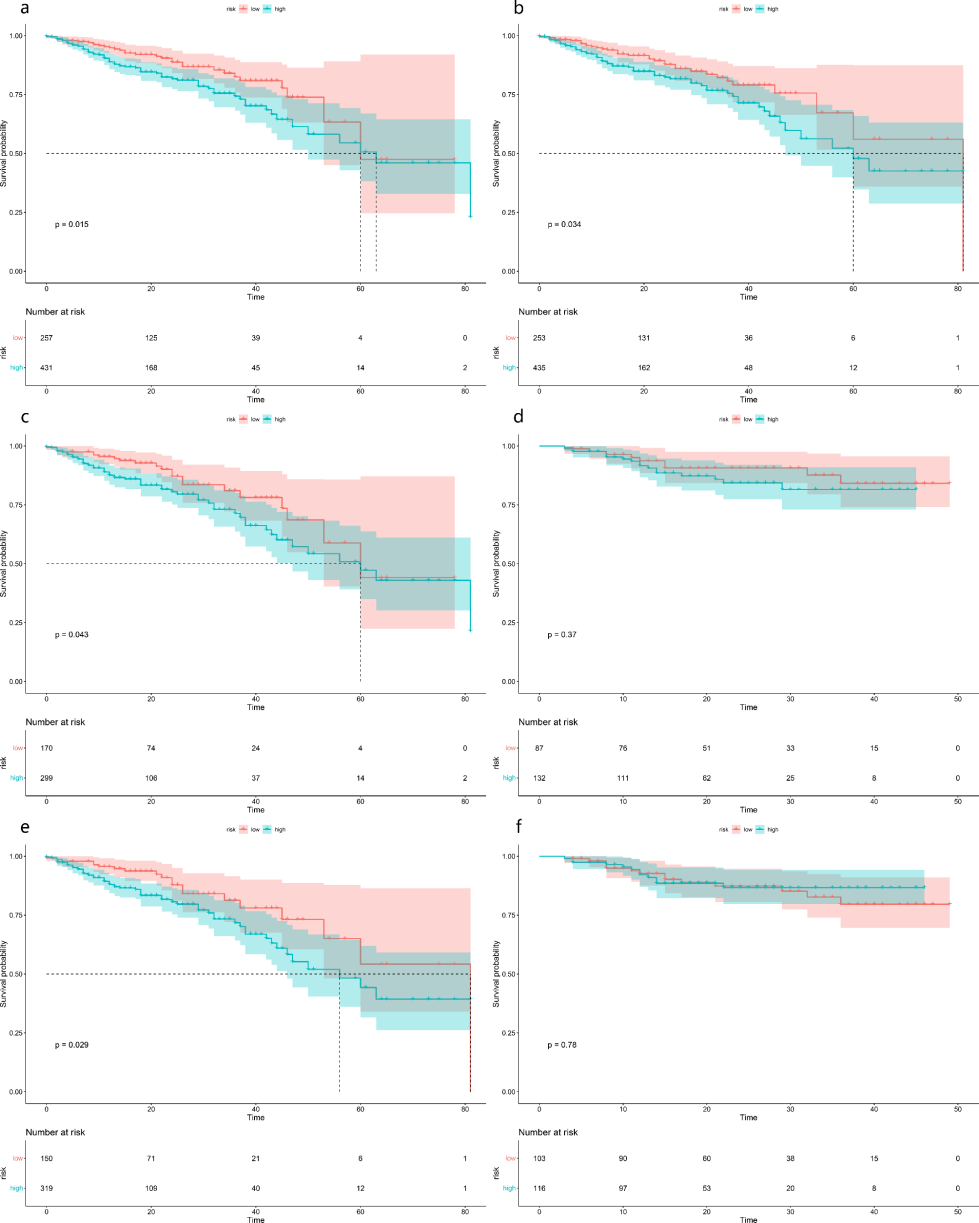
Survival analysis of the pathological report grading model **(a)** and the deep learning radiomics nomogram **(b)**. Survival analysis of the pathological report grading model **(c, d)** and the deep learning radiomics nomogram **(e, f)** for training and external test cohorts

The DLRN was built using the clinical model and the signature from the DLR-based SVM classifier (Fig. 3c). Its prediction efficiency is shown in Table 4. DLRN outperformed both the optimal machine learning model and the clinical model. Figure 4 shows calibration curves and DCA of DLRN. They reveal good calibration and clinical utility of this nomogram. Figure 5 shows Kaplan-Meier survival curves of DLRN for predicting PFS in BCa patients. For the total cohort and training cohort, the pathological report grading model and DLRN could significantly stratify patients for PFS, however this was not the case for the external test cohort.

**Table 4:** Predictive performance of clinical model, the optimal machine learning model, and DLRN.

Model	Training cohort		External test cohort	
	AUC (95% CI)	Accuracy	AUC (95% CI)	Accuracy
Clinical model	0.752(0.707–0.797)	0.723	0.745(0.678–0.813)	0.708
Optimal ML model	0.953(0.931–0.974)	0.902	0.943(0.916–0.970)	0.840
DLRN	0.961(0.944–0.979)	0.919	0.947(0.921–0.973)	0.854

AUC, area under the curve; CI, confidence interval; ML, machine learning; DLRN, deep learning radiomics nomogram

## Discussion

In this study, we built and tested a nomogram based on CT radiomic features that combined clinical features with the DLR signature for prediction of BCa pathological grade. Compared with the clinical model and the radiomics signature, DLRN was associated with superior diagnostic capability (AUC values of 0.961 and 0.947, accuracy values of 0.919 and 0.854 for training and external test cohorts, respectively). This nomogram demonstrated good calibration ability and clinical benefits, indicating that it may represent a helpful preoperative tool for clinical decision making. In addition, the nomogram showed good stratification ability for PFS.

Tumor grade is a critical element for determining therapy and prognosis [22]. This element is crucial for the choice of treatment modality in BCa patients. The results obtained via biopsy sometimes misrepresent the tumor [9], producing a lower grade than the actual grade of the lesion. By integrating the DLR signature with clinical features, DLRN presents substantial potential for identifying the pathological grade of BCa preoperatively, and for aiding patient management.

In current clinical practice, MRI is widely used to evaluate BCa as a noninvasive imaging tool. A previous study [23] used radiomics based on diffusion-weighted imaging and apparent diffusion coefficient maps to grade BCa. This study pointed out that a radiomics strategy may improve BCa grading preoperatively. However, the study did not assess a validation cohort. Wang et al. [9] constructed a multiparametric MRI-based radiomics method, which showed good performance when applied to the validation group (AUC values of 0.9186–0.9276). However, this study relied on a small sample size. Zheng et al. [24] used the radiomics signature and the Vesical Imaging-Reporting and Data System score to construct a nomogram for BCa grading. Their nomogram showed good diagnostic ability (AUC values of 0.956 and 0.958 for training and validation sets, respectively). Additionally, a previous study [25] showed that it is feasible to apply CT texture analysis to identify BCa grading. Zhang et al. [13] built a radiomics model based on CT images for predicting BCa grading, which achieved a fairly good diagnostic efficiency when applied to both training (AUC = 0.950) and validation groups (AUC = 0.860). However, the studies mentioned above were single-center studies without external validation, and only focused on HCR features from imaging.

**Fig. 6:**
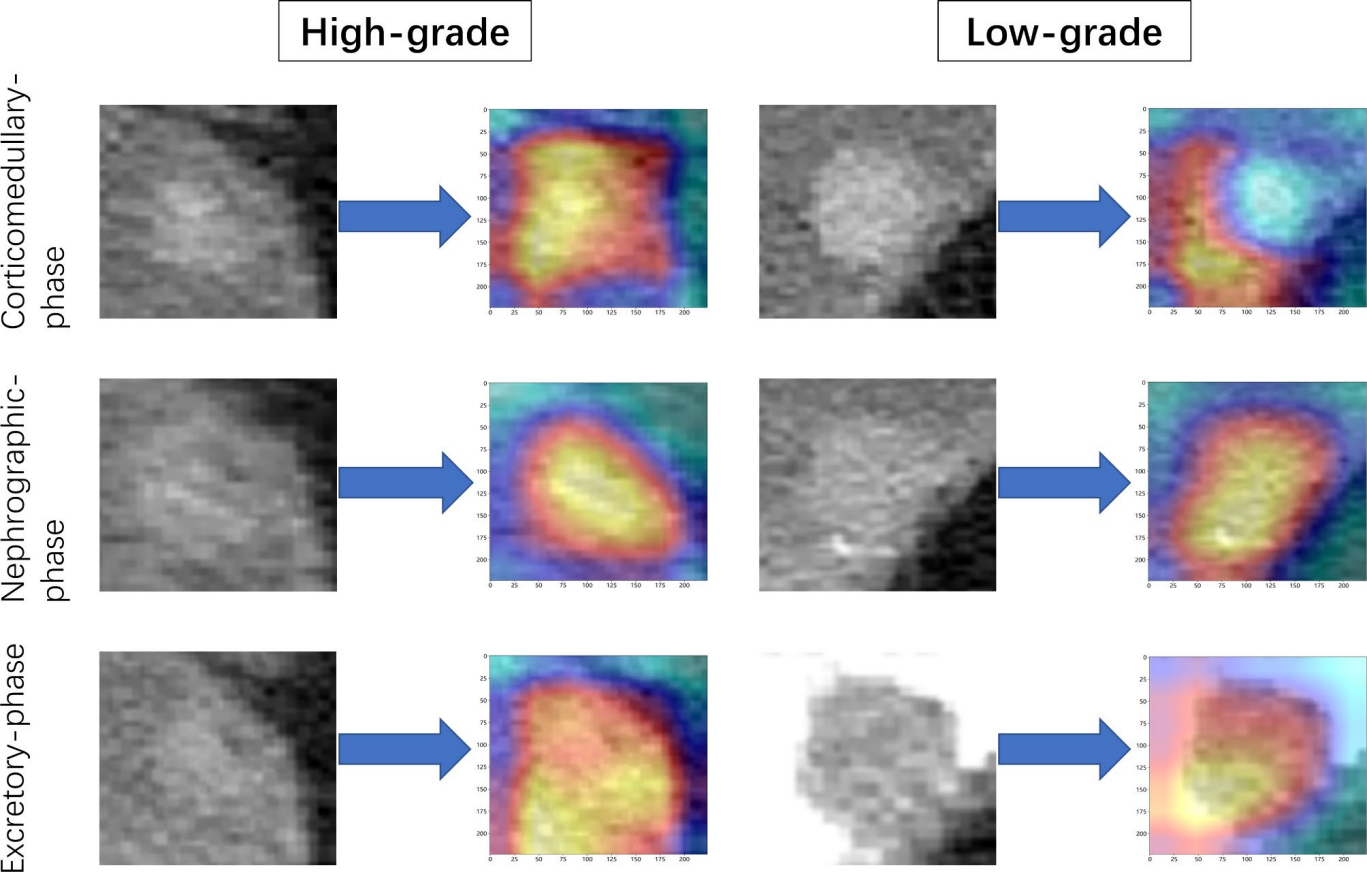
Activation maps of the deep convolutional neural networks for high versus low grade bladder cancer, reflected the weights corresponding to different pathological grades of tumors. These maps were constructed using data from the corticomedullary phase, nephrographic phase, and excretory phase. Red areas indicate higher correlation with tumor grade

DL is a new technique for image analysis. Several studies [18, 19, 26] have demonstrated the value of DL models based on CT image in diagnosis and management of BCa. Compared with previous studies, we extracted DL features to explore the feasibility of applying a DL approach for distinguishing between high and low grade BCa, and then combined DL features with HCR features to predict BCa grading. Among the 60 features screened in this study, including 25 HCR features and 35 DL features, the DL features carried the largest weight, suggesting that the DL technique may have extracted quantitative information reflecting BCa grade. The CNN activation maps contained important regions related to tumor grade. These regions can be used to identify tumor grade via their association with high-grade tumors, and their suppression for low-grade tumors (Fig. 6). Among the selected 25 HCR features, wavelet features accounted for the largest proportion (20/25), and “wavelet_ngtdm ” feature carried the largest weight. Wavelet features could be used to reflect tumor heterogeneity through multi-scale wavelet transform, overcoming the limitations of visual inspection [27]. A previous study [28] showed that wavelet-based features can support tumor grade classification. The diagnostic capability of the model reliant on both HCR features and DL features was superior to that associated with models that relied on HCR features or DL features separately. The signature based on DL features was superior to that based on HCR features, demonstrating the value of the DL approach for grading BCa.

In this study, we used three-phase CT images (including C-phase, N-phase, and E-phase), and collected almost all available CT information from patients. In terms of feature selection, LASSO carries the advantages of ridge regression analysis and good subset screening performance [29]. Efficient and reliable machine learning methods are helpful for promoting the successful application of radiomics in clinical practice, and it is critically important to identify the best machine learning approaches for radiomics strategy [30]. We selected 11 classifier algorithms to increase the reliability of our study (LR, NaiveBayes, SVM, KNN, RandomForest, ExtraTrees, XGBoost, LightGBM, GradientBoosting, AdaBoost, and MLP). SVM algorithm can generally provide better classification because it utilizes the available information to achieve the optimal results, and seems to be superior to a generalization capability when dealing with unseen data [31]. The results of this study showed that the signature from the DLR-based SVM classifier produced the largest AUC and accuracy values among all machine learning models. This was the best machine learning model in our study.

We also evaluated the capability of the DLRN model for predicting the prognosis of BCa patients. High-grade BCa carries a poor prognosis, and higher tumor grade is an independent risk factor for cancer-specific survival in BCa patients [3, 32]. Previous studies have demonstrated the value of radiomic features for survival analysis of BCa patients. Zhang et al. [33] indicated that the radiomics signature was independently associated with PFS in BCa patients. Piotr et al. [34] found that preoperative CT-based radiomic features could predict overall survival of BCa. We found that the pathological report grading model and DLRN presented good prognostic risk stratification capability in all patients, indicating that the proposed DLRN may carry substantial potential for aiding long-term management of BCa patients. The pathological report grading model and DLRN present significant differences in risk stratification when applied to the training cohort, but not with respect to the external test cohort. In all patients, the median PFS times of high-risk and low-risk patients predicted by the pathological report grading model were 63 months and 60 months, respectively. The external test cohort did not include any patient with PFS > 50 months. In addition, there were 61 patients with PFS ≥ 40 months in the training cohort, but only 23 patients with similar PFS values in the external test cohort. These results suggest that the follow-up time of the external test cohort may have been relatively short. In turn, this means that our study was subject to group bias, and that patients in the total cohort and training cohort may better reflect the real PFS of patients.

Our study presents some additional limitations. First, because of its retrospective nature, this study may be affected by selection bias. Therefore, prospective studies will be necessary to verify the predictive capability of our model. Second, this study relied on manual segmentation of tumors, which involved substantial differences across observers. A recent study [35] showed that automatic segmentation achieved good performance. We therefore plan to adopt automatic segmentation in the future. Third, this study only included some baseline clinical data of patients, and the remaining clinical data and biochemical indicators were not collected. Additionally, the MRI data of patients were not analyzed. In the future, it will be necessary to develop a complex prediction model with multi-dimensional information.

## Conclusion

In summary, our DLRN model successfully predicted the pathological grade of BCa and may provide strong support for the development of individualized treatment plans for BCa patients.

### Electronic supplementary material

Below is the link to the electronic supplementary material.

Supplementary Material 1. Additional file 1: table S1 Settings adopted for CT scanning at the three medical centers. Table S2. Performance of different machine learning algorithms with reference to HCR and DL signatures

## Data Availability

The datasets used and/or analysed during the current study are available from the corresponding author on reasonable request.

## Abbreviations

BCa: Bladder cancer
CT: Computed tomography
CTU: CT urography
CNN: Convolutional neural networks
C-phase: Corticomedullary-phase
DL: Deep learning
DCA: Decision curve analysis
DLRN: Deep learning radiomics nomogram
DLR model: Model combining DL features and HCR features
E-phase: Excretory-phase
ExtraTrees: Extremely randomized trees
Glcm: Gray-level co-occurrence matrix
Glrlm: Gray-level run-length matrix
Glszm: Gray-level size zone matrix
Gldm: Gray-level dependence matrix
HCR: Handcrafted radiomics
KNN: K nearest neighbor
LCTV-C: Lesion CT value in corticomedullary-phase
LCTV-N: Lesion CT value in nephrographic-phase
LCTV-E: Lesion CT value in excretory-phase
LightGBM: Light Gradient Boosting Machine
LR: Logistic regression
MLP: Multi-Layer perceptron
N-phase: Nephrographic-phase
Ngtdm: Neighboring grey tone difference matrix
PFS: Progression-free survival
SVM: Support vector machine
TURBT: Transurethral resection of bladder tumor
XGBoost: EXtreme Gradient Boosting
