# Supplementary material for: CT-based deep learning radiomics nomogram for the prediction of pathological grade in bladder cancer: a multicenter study
Source: Cancer Imaging. 2023 Sep 18;23:89. doi: 10.1186/s40644-023-00609-z (PMC10507832; doi:10.1186/s40644-023-00609-z)
Supplement: Supplementary file 1 — Supplementary Material 1. Additional file 1: table S1 Settings adopted for CT scanning at the three medical centers. Table S2. Performance of different machine learning algorithms with reference to HCR and DL signatures [file 40644_2023_609_MOESM1_ESM.docx]

**Table S1** Settings adopted for CT scanning at the three medical centers.

| Parameters | Affiliated Hospital of Qingdao University | Shandong Provincial Hospital Affiliated to Shandong First Medical University | Puyang Oilfield General Hospital |
| --- | --- | --- | --- |
| CT version | Aquilion ONE 640, TOSHIBA; Discovery 750, GE Healthcare; Somatom Sensation Cardiac 64, Siemens Healthcare | Aquilion ONE, TOSHIBA; Discovery 750, GE Healthcare; Somatom Definition Flash, Siemens Healthcare | Optima CT540, GE Healthcare; Brilliance CT, Philips |
| Tube voltage | 120kV | 120kV | 120kV |
| Tube current | automatic tube current modulation | automatic tube current modulation | automatic tube current modulation or 300mAs |
| Detector collimation | 64×0.6mm  or 64×0.625mm | 64×0.6mm  or 64×0.625mm | 64×0.625mm |
| Image matrix | 512×512 | 512×512 | 512×512 |
| Pitch | 0.9 | 0.9 | 0.9 |
| Contrast material | Ultravist 370  or Omnipaque 350 | Omnipaque 350 | Ultravist 370  or Omnipaque 350 |
| Contrast medium dose | 80ml | 80~90ml | 80ml |
| Injection rate | 3.0ml/s | 3.0ml/s | 3.0ml/s |
| Reconstruction slice thickness | 5mm | 5mm | 5mm |

**Table S2** Performance of different machine learning algorithms with reference to HCR and DL signatures.

| **Model** | **Training cohort** | | **External test cohort** | |
| --- | --- | --- | --- | --- |
|  | **AUC (95% CI)** | **Accuracy** | **AUC (95% CI)** | **Accuracy** |
| **HCR signature** | | | | |
| LR | 0.812(0.772-0.851) | 0.742 | 0.846(0.793-0.899) | 0.781 |
| NaiveBayes | 0.766(0.721-0.810) | 0.744 | 0.844(0.791-0.896) | 0.763 |
| SVM | 0.888(0.855-0.921) | 0.810 | 0.803(0.739-0.868) | 0.767 |
| KNN | 0.877(0.848-0.906) | 0.800 | 0.755(0.690-0.819) | 0.717 |
| RandomForest | 0.998(0.996-1.000) | 0.974 | 0.701(0.632-0.771) | 0.644 |
| ExtraTrees | 1.000(nan-nan) | 1.000 | 0.800(0.739-0.861) | 0.772 |
| XGBoost | 1.000(1.000-1.000) | 0.991 | 0.780(0.717-0.843) | 0.731 |
| LightGBM | 0.950(0.931-0.968) | 0.846 | 0.786(0.723-0.848) | 0.658 |
| GradientBoosting | 0.893(0.863-0.922) | 0.793 | 0.796(0.736-0.856) | 0.717 |
| AdaBoost | 0.838(0.802-0.874) | 0.768 | 0.717(0.648-0.787) | 0.667 |
| MLP | 0.850(0.815-0.885) | 0.780 | 0.848(0.795-0.901) | 0.790 |
| **DL signature** | | | | |
| LR | 0.886(0.856-0.916) | 0.817 | 0.826(0.770-0.882) | 0.758 |
| NaiveBayes | 0.843(0.808-0.878) | 0.778 | 0.882(0.835-0.928) | 0.813 |
| SVM | 0.918(0.891-0.945) | 0.844 | 0.875(0.829-0.921) | 0.749 |
| KNN | 0.873(0.843-0.903) | 0.800 | 0.702(0.636-0.769) | 0.653 |
| RandomForest | 0.998(0.996-1.000) | 0.981 | 0.826(0.771-0.881) | 0.735 |
| ExtraTrees | 1.000(nan-nan) | 1.000 | 0.804(0.747-0.862) | 0.726 |
| XGBoost | 1.000(1.000-1.000) | 0.991 | 0.827(0.774-0.879) | 0.744 |
| LightGBM | 0.973(0.960-0.985) | 0.881 | 0.834(0.783-0.886) | 0.726 |
| GradientBoosting | 0.911(0.885-0.938) | 0.808 | 0.833(0.776-0.889) | 0.717 |
| AdaBoost | 0.857(0.824-0.890) | 0.780 | 0.742(0.677-0.808) | 0.717 |
| MLP | 0.912(0.886-0.938) | 0.827 | 0.878(0.834-0.923) | 0.799 |

HCR, handcrafted radiomics; DL, deep learning; AUC, area under the curve; CI, confidence interval; LR, logistic regression; SVM, support vector machine; KNN, K nearest neighbor; ExtraTrees, extremely randomized trees; XGBoost, eXtreme Gradient Boosting; LightGBM, Light Gradient Boosting Machine; MLP, Multi-Layer perceptron.
